# Environmental Surveillance of Polioviruses in Rio de Janeiro, Brazil, in Support to the Activities of Global Polio Eradication Initiative

**DOI:** 10.1007/s12560-015-9221-5

**Published:** 2015-11-04

**Authors:** Joseane Simone de Oliveira Pereira, Lidiane Rodrigues da Silva, Amanda de Meireles Nunes, Silas de Souza Oliveira, Eliane Veiga da Costa, Edson Elias da Silva

**Affiliations:** Enterovirus Laboratory, Oswaldo Cruz Institute, Avenida Brasil 4365, Manguinhos, Rio de Janeiro, RJ CEP 21040-360 Brazil

**Keywords:** Poliovirus, Poliomyelitis, Environmental surveillance, Polio eradication

## Abstract

Wild polioviruses still remain endemic in three countries (Afghanistan, Pakistan, and Nigeria) and re-emergency of wild polio has been reported in previously polio-free countries. Environmental surveillance has been used as a supplementary tool in monitoring the circulation of wild poliovirus (PVs) and/or vaccine-derived PVs even in the absence of acute flaccid paralysis cases. This study aimed to monitor the presence of polioviruses in wastewater samples collected at one wastewater treatment plant located in the municipality of Rio de Janeiro, Brazil. From December 2011 to June 2012 and from September to December 2012, 31 samples were collected and processed. RD and L20B cell cultures were able to isolate PVs and non-polio enteroviruses in 27/31 samples. Polioviruses were isolated in eight samples (type 1 Sabin = 1, type 2 Sabin = 5, and type 3 Sabin = 2). Vaccine-derived polioviruses were not detected nor evidence of recombination with other PVs or non-polio enterovirus serotypes were observed among the isolates. The Sabin-related serotypes 2 and 3 presented nucleotide substitutions in positions associated with the neurovirulent phenotype at the 5′-UTR. Changes in important Amino acid residues at VP1 were also observed in the serotypes 2 and 3. Environmental surveillance has been used successfully in monitoring the circulation of PVs and non-polio enteroviruses and it is of crucial importance in the final stages of the WHO global polio eradication initiative. Our results show the continuous circulation of Sabin-like PVs and non-polio enteroviruses in the analyzed area during the study period.

## Introduction

Poliomyelitis (acute anterior poliomyelitis and infantile paralysis) is characterized by a clinical feature of flaccid paralysis of sudden onset (http://www.who.int/mediacentre/factsheets/fs114/en/). The disease mainly affects children under five years of age and is caused by one of the three poliovirus (PVs) serotypes (PV1, PV2, and PV3) (Pallansch and Roos 2001), which belong to the *Enterovirus* genus of the family *Picornaviridae* (Racaniello 2001; Wimmer et al. 1993).

The global polio eradication initiative was launched in 1988 (CDC 1993; Dowdle et al. 2003; Kew et al. 2005). Since then the incidence of wild PVs transmission has dramatically declined (>99 %) (GPEI 2015). However, by March of 2015, wild polioviruses still remain endemic in three countries (Afghanistan, Pakistan, and Nigeria). Until PVs transmission is interrupted in these countries, all countries remain at risk of importation of polio. In fact, cases of re-emergency have been reported in previously polio-free countries (GPEI 2015).

In the process of eradication, the oral polio vaccine (OPV) has played a crucial role, both by its ease in administration, favoring high vaccine coverage rates, as well as by greater spread of the vaccine virus via the fecal–oral (Carvalho and Weckx 2006). However, as genetically unstable genomes, the attenuated PVs phenotypes present in the OPV may suffer mutations giving rise to rare cases of vaccine-associated poliomyelitis as well as vaccine-derived polioviruses (VDPV) (Kew et al. 2005).

Non-synonymous mutations that may occur at the VP1 gene may change amino acid residues at codons recognized to be associated with the attenuated phenotype of prototype strains. The amino acid residues known to be responsible for OPV attenuation markers are A^88^ residues T^106^ and F^134^ in Sabin serotype 1 (Rouchaud et al. 1995); the residue I^143^ Sabin serotype 2 (Macadam et al. 1993) and the residue T^6^ in Sabin serotype 3 (Weeks-Levy et al. 1991; Tatem et al. 1992). Several mutations lead to the replacement of amino acid I → T in serotype 2; however, other changes in the same amino acid has been observed in smaller proportions (I → V, I and S → I → N) (Mueller et al. 2009).

Another well-characterized attenuating mutations in the Sabin strains are mutations located in the 5′-untranslated region (5′-UTR) (Minor 1992). These mutations have been identified in PVs of Sabin type 3 (472U→C) (Cann et al. 1984), type 2 (481A→G) (Macadam et al. 1993), and type 1 (480G→A and 525U→C) (Otelea et al. 1993) and are believed to selectively affect initiation of translation of viral polyprotein in neuronal cells (Svitkin et al. 1990; Guest et al. 2004).

Environmental surveillance of polioviruses has been used as a supplementary tool in monitoring the circulation of wild PVs and/or VDPV in environmental samples supposedly contaminated by human feces, even in the absence of reported AFP cases (Hovi 2006; Hovi et al. 2012; WHO 2003).

The last paralytic case caused by wild indigenous PVs occurred in Brazil in 1989. Although Brazil has maintained high rates of OPV coverage (>95 %) (MS 2013), the non-occurrence of poliomyelitis caused by wild polioviruses is not sufficient to reject the risk of reintroduction of wild polioviruses from endemic regions. Therefore, environmental surveillance of PVs is essential in order to detect and monitor the circulation of wild PVs and/or VDPVs even in the absence of reported AFP cases (WHO 2003).

The present study aimed to establish the environmental surveillance of PVs in the Enterovirus Laboratory, FIOCRUZ, Brazil (WHO Regional Reference Laboratory) in support to the WHO polio global eradication initiative, in the city of Rio de Janeiro, Brazil.

## Materials and Methods

### Sample Collection

Rio de Janeiro city is one of the 92 municipalities of the Rio de Janeiro State, located in the Southeastern Brazil. This municipality has an area of 1200 km^2^ with approximately 6453,682 inhabitants (IBGE 2014) and is served by wastewater treatment plants (WWTPs). Among the WWTPs, the Alegria/Cedae was chosen because it attends a population of about 1,500,000 inhabitants.

From December 2011 to June 2012 and from September to December 2012, a total of 31 composite samples were collected from the WWTP Alegria/CEDAE once a week. Several aliquots were collected by the grab method during a 12 h period in a total volume of 1.0 L of sewage sample. Samples were transported at 4 to 8 °C to the Laboratory.

### Sample Preparation

Sewage samples were divided into two aliquots of 500 ml each. An aliquot of 500 ml was stored at −20 °C as a backup while the other one was concentrated (100-fold) to 5 ml using the silica-adsorption method (Boom et al. 1990; Leisinger and Metzler 1997; Baggi et al. 2001; van Heerden et al. 2005) essentially as detailed by Zurbriggen et al. (2008). The concentrate was treated with antibiotics and stored at −70 °C before being inoculated in cell cultures.

### Virus Isolation

Each concentrate sample was inoculated (0.5 ml/flask) in 2 L20B and 1 RD cells flasks (25 cm^2^) using standard operating procedures (WHO 2003; WHO 2004a). The flasks were incubated for 7 days at 36 °C. Second passages were performed by equal period.

### Identification of the Isolates

Virus isolates were confirmed as enteroviruses by one-step RT-PCR assay, using specific broad reactive primers EVF = 5′-CTC CGG CCC CTG AAT GCG GCT A-3′ and EVR = 5′-ATT GTC ACC ATA AGC AGC C-3′, conserved in genomes of all known human enteroviruses, as described (dos Santos et al. 2012). This pair of primers is used routinely in the Enterovirus Laboratory for the molecular diagnosis of enteroviruses. Briefly four microliters of the cell culture suspension containing the isolated viruses were added to the PCR mix, composed of 50 pmol of each primer, 12.5 μl of GoTaq Green Master Mix (Promega, Fitchburg, WI, USA), and PCR water to a final volume of 25 μl. PCR was performed with a prior denaturation step of 3 min at 95 °C and 35 cycles of 45 s at 95 °C, 45 s at 55 °C, and 45 s at 70 °C, with a final extension of 7 min at 70 °C in a thermocycler GeneAmp PCR System 9700 (Applied Biosystems, Foster City, CA, USA). The visualization of the PCR amplified products was done by electrophoresis on 10 % acrylamide gels, using the 50 bp marker (Invitrogen, Carlsbad, CA, USA), staining with 0.1 μg/ml ethidium bromide. PVs isolates were identified as such by primers panPV PCR-1 5′-T TIAIIGC(A/G)TGICC(A/G)TT(A/G)TT-3′; panPV PCR-2 5′-CITAITCI(A/C)GITT(C/T)GA(C/T)ATG-3′ and at the serotype level using the sets seroPV1,2S 5′-TGCGIGA(C/T)ACIACICA(C/T)AT-3′; seroPV1A 5′-ATCATICT(C/T)TCIA(A/G)CAT(C/T)TG-3′; seroPV2A, 5′-A(C/T)ICC(C/T)TCIACI(A/G)CICC(C/T)TC-3′; seroPV3S, 5′-AA(C/T)CCITCI(A/G)TITT(C/T)TA(C/T)AC-3′; and seroPV3A, 5′-CCIAI(C/T)TGITC(A/G)TTIG(C/T)(A/G)TC-3′, described elsewhere (Kilpatrick et al. 1996, 1998, 2009) as recommended by WHO (WHO 2004b).

### Genomic Characterization of the Poliovirus Isolates

PVs isolates were characterized as vaccine or wild strains by multiplex RT-PCR, using a PVs Diagnostic PCR Kit (CDC, Atlanta, GA, USA) containing Sabin-specific primers (Sabin1R = 5′-TCCACTGGCITCAGTGTT-3′; Sabin1F = 5′-AGGTCAGATGCTTGAAAGC-3′; Sabin2R = 5′-CGGCTTGTGTCCAGGC-3; Sabin2F = 5′~CCGTTGAAGGGATTACTAAA-3′; Sabin3R = 5′-TAAGCTATCCTGTTGCC-3′; and Sabin3F = 5′-AGGGCGCCCTAACIYTG-3′ as described by Yang et al. (1991). 5′-AGGGCGCCCTAACIYTG-3′. Programmed amplification cycles: thirty cycles of denaturation: 94 °C/30 s; annealing: 62 °C/45 s; extension: 72 °C/1 min in a GoTaq Green Master Mix (Promega, Fitchburg, WI, USA) buffer system. Reaction products were visualized in polyacrylamide gel at 10 %, staining with 0.1 μg/ml ethidium bromide, according to WHO Guidelines (WHO 2004b).

All PVs isolates were examined in the entire VP1 gene to score the presence of mutations, the 5′-UTR for the identification of the three-nucleotide positions associated with neurovirulence (nt 480 for Sabin 1, nt 481 for Sabin 2 and nt 472 for Sabin 3) and, 2C and 3D genes for the presence of genome recombination according to conditions described by Kilpatrick et al. (2004).

### Viral RNA Extraction and Sequencing Reactions

Viral RNA was extracted from cell culture supernatant using QIAamp Viral RNA Mini Kit (Qiagen, Santa Clara, CA, USA), according to the protocol provided by the manufacturer and the cDNA was synthesized in a 20 μl mixture containing 10 μl of the extracted RNA, 50 ng of randon-primers, dNTP mix at 10 mM each and Superscript II (Life-Technologies). Incubation was at 42 °C for 30 min. The entire VP1 coding region was amplified, using primers Q8 = 5′-AAGAGGTCTCTATTCCACAT-3′, and Y7 = 5′-TTT GTG TCA GCG TGT AAT GAC-3′ (Rico-Hesse et al. 1987) while the 5′-UTR were amplified by the primers EVR = 5′-ATTGTCACCATAAGCAGCC-3′ (dos Santos et al. 2012) and S1-1S (5′-TTAAAACAGCTCTGGGGTTG-3′). Reactions contained 5 μl of cDNA, 50 pmol of each primer, GoTaq Green Master Mix (Promega, Fitchburg, WI, USA), and water to a final volume of 50 μl. Cycling conditions: thirty cycles of denaturation: 94 °C 40 s; annealing: 55 °C/40 s; extension: 72 °C/2 min. Amplified products for VP1 gene (~1100 bp) and 5′-UTR (~700 bp) were analyzed in 1.0 % agarose gel containing ethidium bromide (0.5 mg/ml) and visualized under a UV DNA transilluminator. Cycle-sequencing reactions using the same oligonucleotide primers described above were performed using the ABI BigDye terminator cycle-sequencing ready reaction (PE Applied Biosystems, Foster City, and the sequence products were analyzed using the Hitachi 3730XL DNA Analyzer (Applied Biosystems). In order to analyze the presence of mutations in the VP1 gene and further characterize the PVs isolates as vaccine, VDPV or wild strains, the obtained VP1 sequences were compared with the Sabin 1, 2, and 3 prototype strains, respectively (GenBank accession no AY1842219, AY184220, and AY1842221) using the Blast 2.2.27 Program (Altschul et al. 1990). The same was performed for 5′-UTR sequences for the identification of the three known reversions in the 5′-UTR of the three-PVs strains (nt 480 for Sabin type 1, nt 481 for Sabin type 2, and nt 472 for Sabin type 3).

## Results

### Virus Isolation

The rate of viral isolation in RD and L20B cell lines was 87 % (27/31) distributed as follows: 22 samples showed characteristic enteroviruses CPE only in lineage RD; one sample only in L20B and four samples in RD and L20B, simultaneously (Table 1). All non-polio enteroviruses were isolated only on RD lineage while PVs strains were isolated in RD and L20B: RD = 3 isolates; L20B = 1 isolate and RD/L20B = 4 isolates. The lineage L20B was only able to isolate PVs (Table 1). PV strains were present in eight out of 27 (29.6 %) samples with viral isolation. One sample (3.7 %) was positive for PV1, five (18.5 %) for PV2, and two (7.4 %) for PV3 (Table 2). The remaining isolates were characterized as NPEV.

**Table 1:** Viral isolation in cell lineages RD and L20B from sewage samples

Cells lineages	No. of samples with viral isolation^a^/no. of samples tested (%)	No. of poliovirus detected/no. of samples with viral isolation (%)
RD	22/31 (70.9)	03/22 (13.6)
L20B	01/31 (3.2)	01/01 (100)
RD + L20B	04/31 (12.9)	04/04 (100)
Total	27/31 (87)	08/27 (29.6)

Isolation of enteroviruses and polioviruses in RD and/or L20B cells. Sewage sample concentrates were inoculated in cell flasks (25 cm^2^)

^a^Samples with characteristic enteroviruses cytopathogenic effect. Total of samples tested

**Table 2:** Nucleotide and amino acid substitutions observed within the VP1 gene of poliovirus isolates

Sample no.	Serotype isolated	No. of mutations in VP1	Nucleotide		Amino acid	
			Type of substitutions	Nucleotide substitutions at position^a^	Position of AA in VP1	Change to AA
05	PV2 *Sabin like*	01	NS	A2626G	49	T → A
06	PV2 *Sabin like*	02	NS	T2548C	23	S → P
			NS	T2909C	143	I → T
11	PV2 *Sabin like*	01	NS	T2909C	143	I → T
12	PV2 *Sabin like*	01	NS	T2909A	143	I → N
13	PV3 *Sabin like*	05	NS	C2493T	6	T → I
			S	C2683T	69	–
			S	A2698G	74	–
			S	A2821G	115	–
			NS	C2967T	164	T → I
15	PV1 *Sabin like*	02	NS	A2774G	295	K → E
			NS	A3059T	194	I → F
16	PV2 *Sabin like*	02	NS	T2909A	143	I → N
			S	A3363G	294	–
23	PV3 *Sabin like*	03	NS	C2493T	6	T → I
			S	C2683T	69	–
			S	A2869G	131	–

^a^Left-hand letter refers to Sabin original nucleotide; right-hand letter refers to nucleotide present on the isolated strain

*S* synonymous mutations, *NS* non-synonymous mutations, *AA* amino acid, – no change AA, *A* alanine, *E* glutamic acid, *F* phenylalanine, *I* isoleucine, *K* lysine, *N* asparagine, *P* proline, *S* serine, *T* threonine

### Genomic Characterization of the Polioviruses Isolated from the Environment

#### VP1 Analysis

The number of nucleotide substitutions in VP1 varied from 0 to 5, therefore all strains were considered as Sabin-like according to WHO instructions (≤9 nucleotide differences for serotype 1, ≤5 nucleotide differences for serotype 2, and ≤9 nucleotide differences for serotype 3) (Table 2). None of the vaccine-related PVs strains showed a number of mutations sufficient to be classified as VDPV.

All Sabin-related PVs strains showed at least one non-synonymous mutation. The sole isolate of serotype 1 presented two non-synonymous mutations (K^295^ and I^194^) not located in amino acid residues considered as markers of attenuation (Table 2).

Changes at the amino acids at residues I^143^ (I → T) and I^143^ (I → N) were the most frequently mutated codons present in four of the five isolates of serotype 2 while the remaining PV2 showed a non-synonymous mutation causing an amino acid change at residue T^49^ (T → A) (Table 2).

The two PV3 isolates showed non-synonymous mutations at nucleotide position C2493T leading to amino acid changes at residue T^6^ (T → I) of VP1. One of them showed the non-synonymous mutation at nucleotide C2967T with amino acid change at position T^164^ (T → I) (Table 2).

### 5′-UTR Analysis

No substitution was identified for the Sabin type 1 isolate at the position 480. Four out of five Sabin 2 isolates showed the substitution A481G, associated to the neurovirulent phenotype while only one of the Sabin 3 isolates presented the substitution U472C, also associated with neurovirulence.

### 2C and 3D Genes Analysis

RT-PCR analysis of the isolates in both 2C protease and 3C polymerase showed no evidence of genome recombination with other PVs or non-polio enterovirus species.

## Discussion

While eradication is not achieved, the circulation of wild strains and VDPVs will continue to challenge the global eradication of polio (Kew et al. 2005; Minor 2009; Roivanem et al. 2010).

In support of the global polio eradication activities in Brazil, this study aimed to isolate and characterize circulating PVs from wastewater collected in a large WWTP attending ~1.5 million inhabitants in Rio de Janeiro city and to establish the environmental surveillance at the Enterovirus RRL/Fiocruz.

In the present study, the rate of polio + non-polio enterovirus isolation in L20B and RD cell lines was 87 %, in agreement with a work by Vinjé et al. (2004), which reported 85 %.

The detection of non-polio enteroviruses in the samples is an indication that the environmental monitoring is being adequately performed because at least 30 % of non-polio enterovirus are expected to be found in samples processed by the Grab method (Lewis and Metcalf 1988; WHO 2003). Accordingly, Sabin-related polioviruses should also be encountered in OPV-vaccinated populations especially during and after national immunization campaigns (WHO 2003).

Vulnerable groups such as immunodeficient individuals exposed or vaccinated with OPV may contribute to the circulation of PVs in the environment for long periods (Kew et al. 2005; Khetsuariani et al. 2010; Lodder et al. 2012). The presence of at least 1 % of divergence in VP1 gene for serotypes 1 and 3 and 0.6 % for serotype 2, in comparison with the prototype strains, classifies them as VDPV. PVs with lower divergences than these are considered “Sabin-like” (Blomqvist et al. 2010). This demarcation of ~1 % difference in nucleotide sequence of VP1 indicates that replication of the vaccine virus has occurred for approximately one year (Kew et al. 2005).

All PVs strains isolated in this study showed few nucleotide differences compared with the prototype vaccine strains. Thus, VDPVs were not detected among the isolates. Similar results were reported by Gregio (2006) studying Sabin-related PVs from wastewater samples collected in São Paulo state, Brazil.

One serotype 2 isolate collected one week after the national immunization campaign held on June 18, 2012, showed the presence of a non-synonymous mutation in the amino acid I^143^ (T → I). It is possible that the referred mutation arose in the early stages of the Sabin type 2 replication. It is suggested, therefore, that the I^143^ substitution present in this isolate has emerged in the early stages of viral replication in the vaccine recipients.

In a previous study analyzing clinical isolates, changes in the amino acid I143 (I → T) for serotype 2 was consistently observed (Costa 2011). It can be deduced that these mutations are common in circulating poliovirus, as it is also observed in PVs isolated from clinical cases and may represent a risk to susceptible individuals since this might represent some degree of loss of attenuation.

Although in small number it was observed that serotype 1 PVs was less frequent (12.5 %–01/08) than serotypes 2 (62.5 %–05/08) and 3 (25 %–02/08), which is consistent with the data reported in New Zealand, by Huang et al. (2005) and Cordoba, Argentina (Mueller et al. 2009). It is believed that this difference may be related to the amount of excreted virus particles; the duration of excretion; the virus stability in the environment or in the ability to be transmitted from vaccine-recipient individuals to their contacts (Mueller et al. 2009).

An environmental monitoring study done by Roivainen et al. (2010) showed that strains of VDPVs were repeatedly detected in sewage in Finland. Although no suspected polio cases have been reported in the country since 1985, the authors of this study emphasized the importance of maintaining high vaccination coverage rates and environmental monitoring (Roivanen et al. 2010).

Due to the present circulation of wild and VDPV’s in some countries of the globe, regions with immunization coverage rates below the desired or non-homogeneous coverage may have groups of susceptible individuals. Therefore, the environmental surveillance of polioviruses is essential during the final stages of the global polio eradication.

## Conclusions

Environmental surveillance has been used successfully in monitoring the circulation of enteroviruses and has crucial importance in the final stages of the WHO global polio eradication initiative. Our results show the continuous circulation of Sabin-like PVs and absence of VDPV’s in the analyzed area during the study period. We can then infer that the local vaccine coverage has been able to maintain the area free of wild and VDPV’s.
